# Incidence and causes of failure in various anatomical pouch designs 20 years after surgical primary ileal-pouch anal anastomosis construction

**DOI:** 10.1007/s00384-022-04280-x

**Published:** 2022-11-11

**Authors:** Maud A. Reijntjes, Eva K. Bocharewicz, Roel Hompes, Christianne J. Buskens, Willem A. Bemelman

**Affiliations:** 1 Department of Surgery, Amsterdam UMC, University of Amsterdam, IBD Unit Ospedale San Raffaele, Meibergdreef 9, 1105 AZ Amsterdam, the Netherlands; 2 Gastroenterology and Endoscopy, IBD Unit, IRCCS Ospedale San Raffaele and University Vita-Salute San Raffaele, Milan, Italy

**Keywords:** Ulcerative colitis, Ileo-pouch anal anastomosis, Pouch design, Long-term outcomes

## Abstract

**Purpose:**

Since the introduction of ileo-pouch anal anastomosis (IPAA) surgery, various pouch designs have been applied. Recently, there has been renewed interest in creating larger pouch designs to reduce defecation frequency after pouch surgery. The aim of this study was to assess chronic pouch failure (PF) rates and causes in alternative S or septated (SP) pouches when compared to J pouches and B- shaped adaptations.

**Methods:**

This retrospective cohort study included patients that underwent primary IPAA construction surgery from 1978–2000. Pouch designs were subdivided in J and B (J/B), and larger pouches (S/SP). PF included need for a pouch excision, redo pouch procedure, revisional pouch surgery, or permanent ileostomy surgery. Outcomes of this study were incidence and causes for PF per pouch design group.

**Results:**

Out of 200 patients who underwent IPAA surgery, 19 had an S/SP design and 181 had a J/B design. After a follow-up of 27.0 (IQR 23.3 – 29.0) years, 45/200 (22.5%) patients who underwent IPAA surgery between 1975–2000 developed PF. Some 78.9% of patients with an S/SP pouch developed PF, compared to 16.7% of patients with a J/B pouch (p < 0.01). Mechanical outlet issues occurred more often in S/SP pouches when compared to J/B (42.1% vs. 1.1%, p < 0.01), and were predominantly caused by septal- or pouch wall intussusception and efferent loop kinking (S-pouch).

**Conclusion:**

Despite an inevitable proportion of bias, the current study revealed that S/SP pouches were characterized by an increased PF incidence due to emptying problems after long-term follow-up when compared to J/B pouches. Constructing an S pouch or large septated reservoir at index surgery should therefore be questioned because of a shorter longevity.

**Supplementary Information:**

The online version contains supplementary material available at 10.1007/s00384-022-04280-x.

## Introduction

**Fig. 1:**
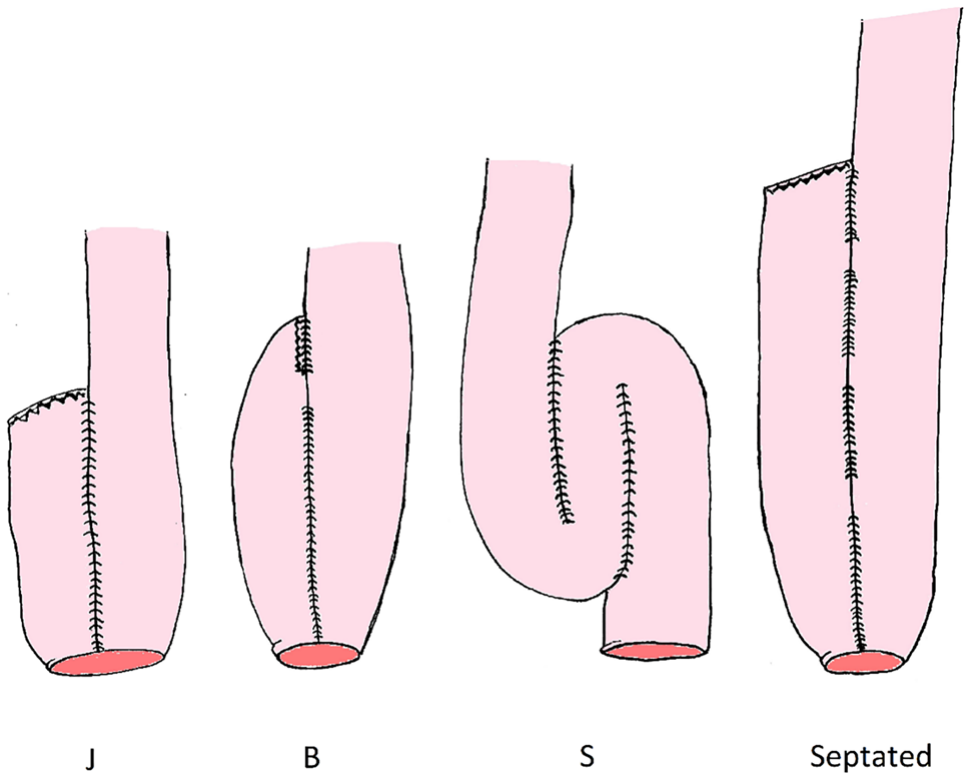
Schematic overview of pouch designs (**a**): J-pouch, (**b**): B-pouch, (**c**): S-pouch, (**d**) Septated pouch

Restorative procto-colectomy with consecutive ileal-pouch anal anastomotic (IPAA) surgery is standard surgical therapy for patients with medical refractory Ulcerative Colitis (UC) and familial adenomatous polyposis (FAP). Various surgical techniques have been proposed in the early days of IPAA surgery. The first pouch-design created in 1978 was the ‘S’ pouch [1]. The body of an S pouch is constructed with two side-to side anastomoses with an end-to end ileoanal anastomosis, resulting in a relatively large pouch. Subsequently, a number of alternative designs (e.g. W, J and B) have been proposed, in search of the most optimal design [2, 3]. The W pouch also has a relatively larger volume as multiple side-to side anastomoses create the pouch body with a side to end ileoanal anastomosis. The J pouch is constructed with a generally smaller pouch volume with a side to end ileoanal anastomosis. The B pouch is an adaptation of the J pouch with the efferent loop anastomosed in the afferent loop. The J pouch is the most common design worldwide since the late 1990’s, primarily due to ease of surgical construction and superior emptying (Fig. 1). The lower volume of J (and B) pouches resulted in a slightly higher defecation frequency when compared to the larger S and W pouches [4–6]. In attempt to ommit this higher defecation frequency, there has been renewed interest in creating larger sized pouches such as the K pouch [7]. However, previous studies concluded that in particular during the first year after IPAA surgery, pouch function is impaired due to a lower compliance as measured using an electronic barostat in J pouches [8]. One year after initial IPAA surgery, the relatively small pouches have matured, resulting in a negligible difference when compared to the larger pouches with respect to number of bowel movements^8^. Although functional outcomes are clinically relevant, long-term outcome parameters should be taken into consideration as well before selecting a pouch design. Besides pouch function and complexity of surgical construction, failure-free pouch survival is a relevant factor in determining the most optimal anatomical pouch design. When it comes to failure-free pouch survival, studies comparing long-term results subdivided by pouch design did not report major differences between S and J pouches [4]. An overall 10-year survival of pouches between 94–96% [9, 10] was reported with a 20-year survival rate of 93% [10]. The objective of this study was to assess pouch failure (PF) in different pouch designs compared according to volume, with a follow up exceeding 20 years. Moreover, we classified causes of PF per pouch design following a sub-classification for causes and corresponding surgical treatment of PF [11]. Defining these outcomes may contribute to identification of the most sustainable pouch design.

## Methods

### Cohort definition

This retrospective cohort study was performed in the Amsterdam University Medical Centre, location AMC, a tertiary refferal centre for pouch surgery. Patients who underwent IPAA pouch surgery between January 1^st^ of 1978 and December 31^st^ of 2000 were included. Patients who underwent primary IPAA surgery after 2000 were excluded as a minimum postoperative follow-up of 20 years was pursued in this study. Patients who underwent primary IPAA surgery elsewhere undergoing redo surgery in the conducting centre were excluded. Patients were asked permission to use their data by opt-out procedure, and the medical ethical committee waived ethical approval for this retrospective study. All procedures, regardless indication for pouch-surgery (i.e. therapy refractory UC, FAP or UC and dysplasia) were included in this study. One stage, (modified) 2- and 3-stage procedures were included in this study.

### Data collection

Patient characteristics, perioperative surgical characteristics and follow-up data were collected from patient reports. Since electronic reports were introduced in 1990, older handwritten reports were obtained from hospital archives. Electronic and archived surgical reports were analysed thoroughly in order to deduct the exact pouch design from the surgical report, with the help of an experienced colorectal surgeon in case of uncertainty. Both archived hospital reports (1979–1990) and electronic patient reports (1990–2000) were inspected. A database was constructed in IBM SPSS version 26.0 containing variables necessary to answer research questions for this study.

### Outcome definitions

The following main outcomes of this study were compared between pouch design groups:

1. PF at end of follow-up
2. Failure-free pouch survival after 10 and 20 years
3. Correlation of pouch failure cause and pouch design
  Pouch failure (PF) was defined as chronic pouch-related complications requiring surgical treatment after a minimum of 3 months following initial IPAA surgery. The following procedures were included in PF:
4. Pouch excision: Surgical resection of primary pouch with concurrent permanent ileostomy.
5. Redo pouch surgery: Surgical resection of primary pouch with maintaining bowel continuity through construction of a new IPAA.
6. Revisional pouch surgery: Surgical modification of primary pouch whereby conserving (a part of) the primary constructed pouch.
7. Permanent defunctioning by ileostomy: Creation of a definitive ileostomy without surgical modification the pouch.

Patients with PF were categorised by different underlying causes and subsequent surgical management options of PF (Appendix 1) [11].

Patients with long-term postoperative complications after primary IPAA surgery were standardly referred back and followed in the conducting centre, and surgical treatments for PF were standardly performed in the conducting centre. Patients who underwent redo surgery within three months after initial IPAA surgery were included in this study in case the ileoanal pouch remained functional. Postoperative follow-up time was calculated starting from the day of construction (1- and modified 2 staged IPAA) of the pouch or closure of the defunctioning ileostomy (2 and 3-staged IPAA). Patients who were impossible to follow were censored at time of last date of follow-up.

### Pouch designs

**Fig. 2:**
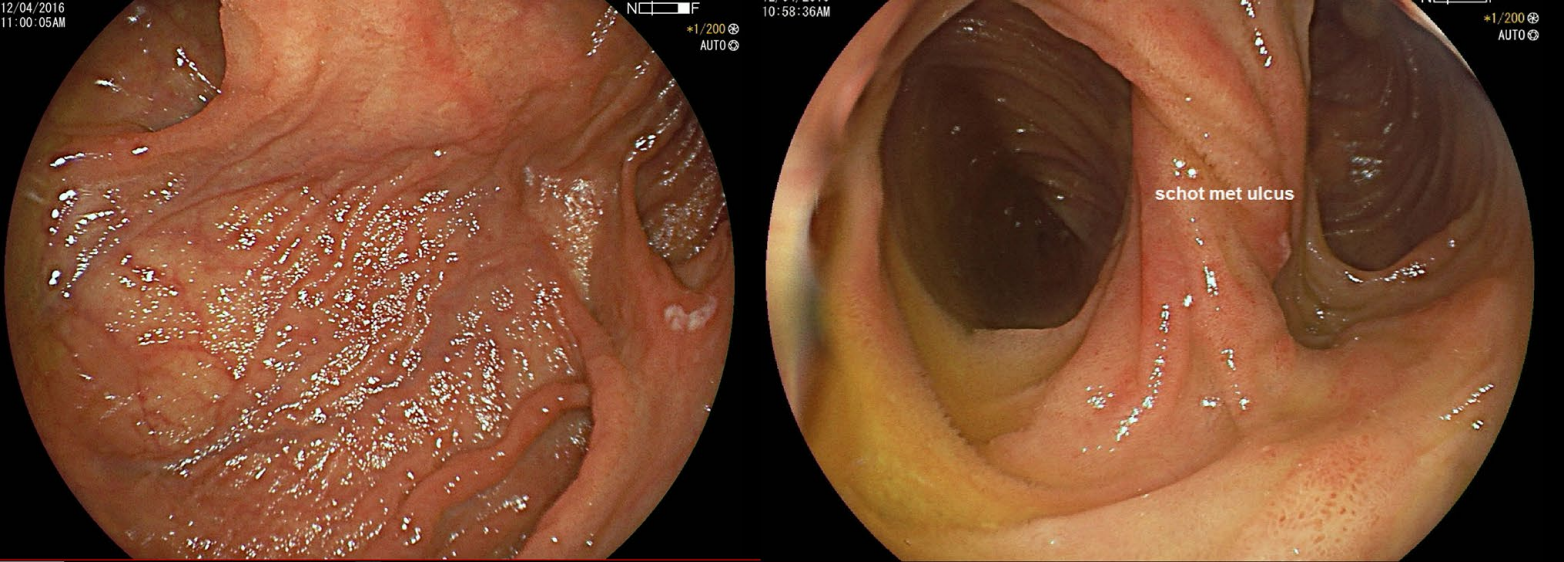
Endoscopy in an SP pouch demonstrating intussuscepting septa surrounded by connecting openings

The following pouch-designs were constructed in our clinical practice: *J-pouch*: In our practice, we construct relatively small pouches of two ileal loops of 10 cm in length. The apex of the pouch is generally stapled to the rectal cuff (Fig. 1a). *B-pouch*: Longitudinal side-to-side anastomosis of 8 cm followed by an end to side anastomosis of the efferent loop into the afferent loop of the pouch. The apex of the pouch is generally stapled to the rectal cuff (Fig. 1b). *S-pouch*: Two parallel longitudinal side-to-side anastomoses with an end-to-end stapled or hand-sewn anastomosis between the terminal ileum and the rectal cuff (Fig. 1c). *Septated pouch*: Long pouch constructed as a J pouch by linear staplers. The staple line was excised and the bowel was reconstructed with manual sutures creating 2–3 septa between side to side anastomoses (Figs. 1d, 2).

As mentioned before, the B pouch is an adaptation of the J pouch. For this reason, J-pouches and B-shaped adaptions were considered as one group during analyses, and were compared to a group containing all remaining designs (S- and septated), according to pouch volume and amount of anastomoses. Pouch-length was mentioned in reports of primary IPAA- procedures, pouch resection specimens, pouch endoscopies or redo pouch procedures. Patients with an unknown pouch-design were excluded from analyses.

### Statistical analysis

Categorical outcomes were expressed as percentage. Continuous variables of secondary outcomes showing normal distribution were expressed as mean with standard deviation (SD) and/or range, continuous variables with no normal distribution were expressed as median with interquartile range (IQR). To compare categorical data, the Chi-square test was used for statistical significance. For comparing continuous data with normal distribution, the independent samples T-test was used. In case continuous data was not normally distributed, the Mann–Whitney U test was used to compare continuous variables. Variables were excluded from statistical analyses if the percentage of missing data exceeded in 30% of all cases. All data were analysed through complete case analyses. Pouch survival over time was described in years, analysed using Kaplan–Meier analysis and compared with the log-rank test. PF-free survival was calculated as the interval (in years) between date of first fecal stream through the pouch and date of PF or date of last follow-up. Date of ileostomy surgery was selected as PF date in case a defunctioning ileostomy without closure preceded the PF procedure. Differences were considered statistically significant if two-sided the *p* values were under 0.05. Statistical analyses were performed using SPSS (IBM Corp., Armonk, NY, USA).

## Results

### Patient demographics

A total of 213 pouches were created between 1979 and 2000. Thirteen patients were excluded as their pouch-design was not specified. The remaining 200 patients underwent IPAA surgery for UC (72.0%), polyposis/malignancy (17.5%), and indeterminate colitis (5%). The majority of IPAA surgeries were performed via a one- (39.4%) or modified two-stage (31.1%) approach. Rectal dissections were performed via posterior meso-rectal excision with anterolateral close rectal dissection.

### Pouch characteristics

A total of 181 (90.5%) pouches had a J (n = 25) or B (n = 148) pouch design. In eight patients, reports did not distinguish J- from B- designs. Nineteen (9.5%) pouches were constructed with an S (n = 2) or septated (SP) design (n = 17). S/SP pouches were constructed earlier when compared to J/B pouches (median operation year 1987 vs 1994). A larger proportion of patients with S/SP pouches underwent IPAA surgery for polyposis (37% vs 12%, p = 0.03) and more patients with an S/SP pouch underwent two- or three- stage IPAA surgery (p < 0.01). Further baseline characteristics are displayed in Table 1.

**Table 1:** Patient demographics

**Patient characteristics**	**Total (n = 200)**		**S/SP design (n = 19)**		**J/B design (n = 181)**			**Missing**
	n %		n %		n %		p-value	n %
**Sex**								
Male	102	51%	12	63%	90	50%	0.3	
**Age at time of IPAA**^**a**^** surgery, median (IQR)**	34 (27.0–42.8)		32.0(23.0–38.0)		34.0 (27.0–43.0)		0.4	
**Year of IPAAa surgery, median (IQR)**	1994 (1991–1997)		1987 (1986–1989)		1994 (1991–1997)		**< 0.01**	
**Pre-IPAA diagnosis**							**0.02**	
UC	144	72%	8	42%	144	80%		
Polyposis/malignancy	35	18%	8	42%	27	15%		
IBD-U	10	5%	3	16%	7	4%		
Other	3	2%	0	0%	3	2%		
**Staged procedure**							**< 0.01**	
One- stage	76	39%	1	6%	75	43%		
Two- stage	34	18%	8	47%	26	15%		8 4%
Modified two-stage	60	31%	3	18%	57	33%		
Three-stage	22	11%	4	24%	18	10%		
Postoperative follow-up, median (IQR), years	27.0 (23.3–29.0)		33.0 (31.0–34.0)		26.0 (23.0–29.0)		**< 0.01**	

Bold values were values with a significance <0.05

^a^*IPAA* Ileal pouch-anal anastomosis

^b^*IQR* interquartile range

^c^*UC* Ulcerative colitis

^d^*IBD-U* IBD-undetermined

Intra-luminal septa were present in SP pouches as a result of creating multiple side-to side anastomoses during pouch construction (Fig. 2). Moreover, longitudinal pouch length was reported in 80 (37.5%) patients. A higher mean longitudinal pouch-length was reported in eight S/SP pouches (25.8 cm, range 14–45) when compared to 72 J/B pouches (13.5 cm, range 9–40, p < 0.01). Pouch-length was mentioned in reports of 69 primary IPAA- procedures, five pouch resection specimens, four pouchoscopies and two redo pouch procedures.

### Pouch failure (PF)

**Fig. 3:**
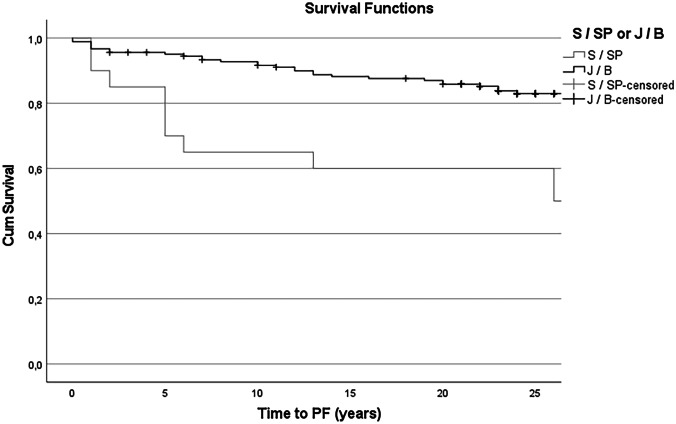
Failure-free pouch survival curve of S/SP pouches (grey) and J/B pouches (black)

The overall median follow-up was 27.0 years (IQR 23.3 – 29.0), with a significantly longer follow-up period for S/SP pouches (33.0 vs 26.0 years, p < 0.01). PF occurred in 45 (22.5%) patients, with a higher PF rate for S/SP pouches when compared to J/B pouches (78.9% vs 16.7%, p < 0.01). This difference persists in consideration of SP pouches only (14/17, 82.4%, p < 0.01) An overall mean failure-free pouch survival of 29.4 (CI 27.6–31.2) years was reported, with a shorter mean failure-free pouch survival for S/SP pouches when compared to J/B pouches (20.1 vs 30.8 years, p < 0.01). Ten- and 20- year survival rates for patients with S/SP pouches compared to J/B pouches were 63.2% and 57.9% vs. 91.6% and 85.8% respectively (p < 0.01). Survival curves are displayed in Fig. 3. The overall median time to PF was 11 (IQR 3.5–22.5) years, with a median time to PF of 13 (IQR 5.0–29.0) years for S/SP pouches and 10.50 (2.0–19.3) years in J/B pouches (p = 0.22).

### Causes of pouch failure

**Fig. 4:**
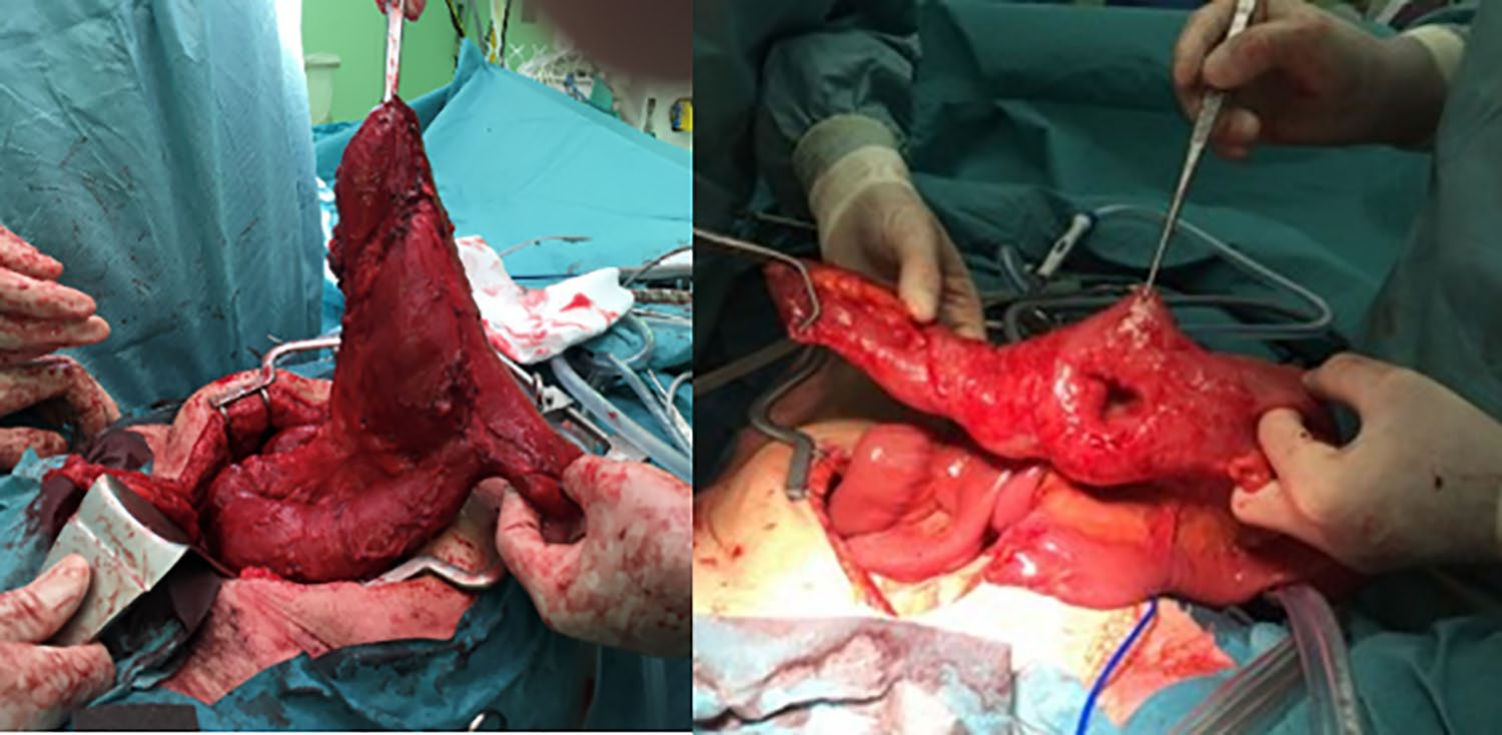
Excisions of a megapouch (a: S design, b: SP design)

**Fig. 5:**
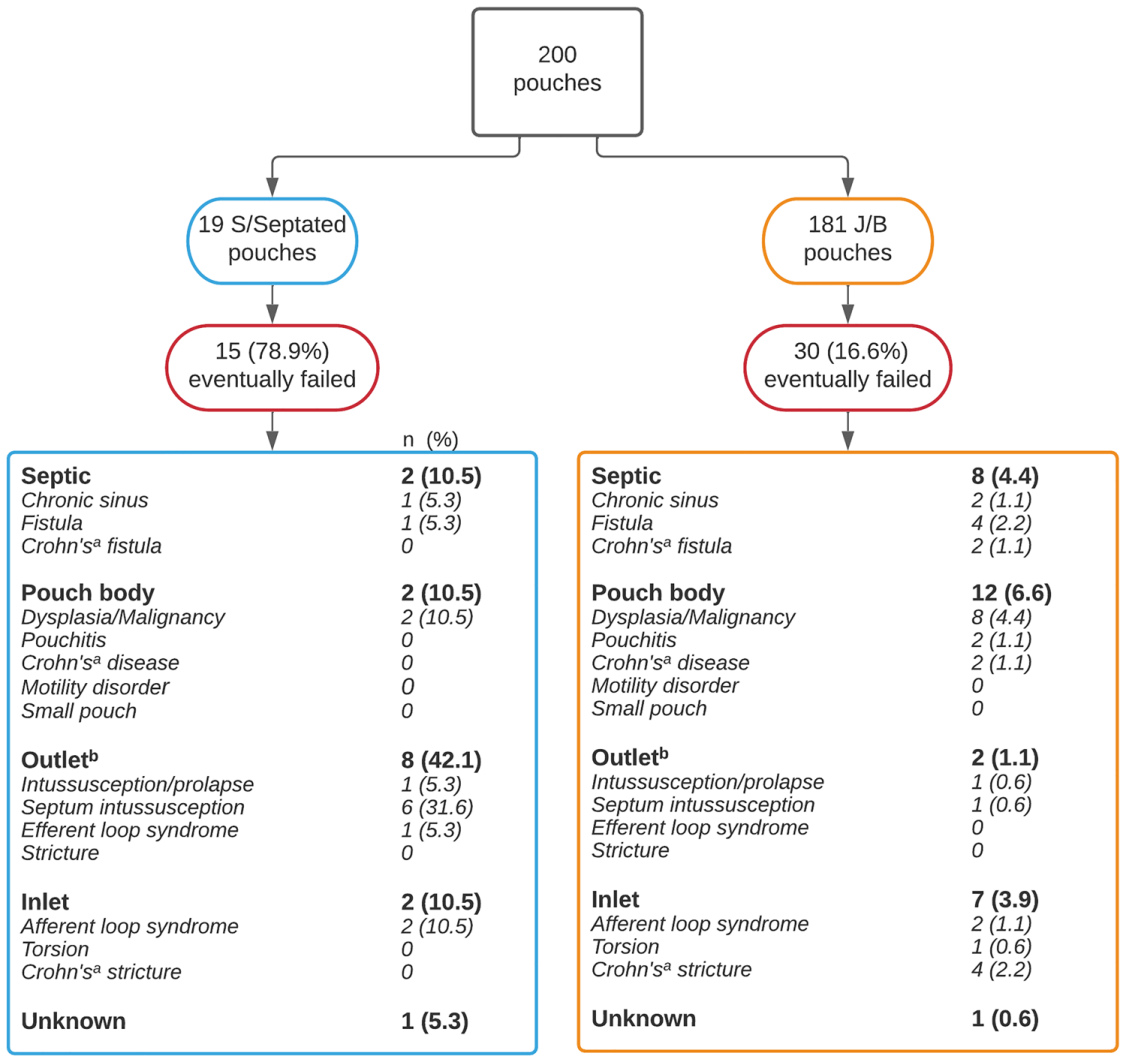
Reasons of PF between S/SP and J/B pouches. n (%): percentages of total amount of corresponding pouch design ^a^Crohn’s disease in the pouch was either histopathologically proven or strongly suspected. ^b^Outlet problems were diagnosed by endoscopy or defecating pouchography and were unresponsive to non-surgical treatment

No differences between S/SP and J/B pouches were reported for incidence of septic- (11% vs. 4.4%, p = 0.24), pouch body- (11% vs. 6.6%, p = 0.63) or inlet issues (11% vs. 3.9%, p = 0.21). A significantly higher proportion of S/SP pouches developed outlet issues (42% vs. 1%, p < 0.01). Four out of the 8 patients with an S/SP design had a megapouch (Fig. 4). Six SP pouches had septal intussusception, and one had pouch wall intussusception. One S pouch developed efferent loop syndrome. Two out of 181 (1.1%) patients with a J/B pouch had outlet issues of which none had a megapouch; intussusception of one proximal septum occurred in a B pouch, and wall intussusception occurred in a J pouch. Other causes of PF per pouch design are demonstrated in Fig. 5. The median time to the surgical procedure for PF ranged from 4 years for septic complications to 26 years for outlet complications (Table 2).

**Table 2:** Outcomes of PF surgery per design

	**Overall**	**S/SP pouches**	**J/B pouches**	**p-value**
**PF**^**a**^** incidence, n (%)**	45 (22.5)	16 (78.9)	29 (16.7)	< 0.01
**10-year failure-free pouch survival, %**	89.9	63.9	91.6	< 0.01
**20-year failure-free pouch survival, %**	84.2	57.9	85.8	
**Pouch size, cm **^**b**^**, mean (range)**	14.7 (9.0–45.0)	25.8 (14.0–45.0)	13.5 (9.0–40.0)	< 0.01
**Time to surgery for PF, years, median (IQR**^**c**^**)** ^d^ *Septic* *Pouch body* *Outlet* *Inlet*	11.0 (3.5–22.5) 4.0 (1.0–4.8) 11.5 (6.5–19.3) 26.0 (5.8–30.3) 11.0 (1.5–23.0)	13.0 (5.0–29.0) 3.5 (1.0-nr) 17.0 (5.0-nr) 26.5 (16.3–30.8) 1.5 (1.0-nr)	10.5 (2.0–19.3) 7.0 (1.3–20.3) 11.5 (7.3–18.3) 3.0 (0.0-nr) 13.0 (7.0–24.0)	0.22 0.42 0.78 0.07 0.14

^a^*PF* Chronic pouch failure

^b^Centimeter

^c^*IQR* interquartile range

^d^A defunctioning ileostomy without closure preceded the PF procedure in three patients

### Surgical therapy for PF

Out of 45 patients with PF, 19 patients underwent revisional pouch surgery for PF, and three underwent a redo pouch procedure. Fourteen patients underwent a pouch excision and nine patients required definitive ileostomy surgery.

### S/SP pouches

Fifteen patients with an S/SP pouch developed PF. One patient with an outlet issue and one patient with recurrent polyposis underwent a redo pouch procedure. Six patients with outlet issues and two patients with inlet issues underwent revisional pouch surgery. One patient with a fistula and one patient with a malignancy in the pouch underwent a pouch excision. One patient underwent a pouch excision for an unknown reason. One patient with an outlet issue and concurrent pouchitis and one patient with a chronic sinus required permanent pouch defunctioning by definitive ileostomy.

### J/B pouches

Thirty patients with a J/B pouch required surgical treatment for PF. One patient with a chronic sinus underwent a redo pouch procedure. Six patients with inlet issues, two patients with septic complications, two patients with outlet issues and one patient with recurrent dysplasia underwent revisional pouch surgery. Seven patients with pouch body issues and three patients with septic complications underwent a pouch excision. One patient underwent a pouch excision for an unknown reason. Four patients with pouch body complications, two patients with septic complications and one patient with afferent loop syndrome required permanent pouch defunctioning by definitive ileostomy.

## Discussion

The current study reports on long-term PF rates and failure-free pouch survival of alternative (S/SP pouches) when compared to the J pouch and B shaped adaptions. We demonstrated an overall 79% PF rate in the S/SP pouches after a median of 13 years. Overall PF rates were relatively high (23%) as pouches in this cohort were created in the early days of pouch surgery. The remarkably high failure rate in the S/SP pouches (79% vs. 17% in J/B design) is mainly the result of mechanical outlet issues, as clarified by the proposed sub-classification demonstrating causes for PF. Kinking of the efferent loop and intussusception in combination with oversizing of the pouch caused these outlet problems [12, 13]. Redo surgery for outlet complications occurred after a relatively long postoperative follow-up. The heterogenuous clinical manifestation of outlet issues however typically causes significant diagnostic and therapeutic delay. Incidence of pouch failure did not demonstrate such remarkable differences between the S/SP and J/B cohorts in relation to septic, pouch body, or mechanical inlet obstructions.

The median shorter postoperative follow-up period for the cohort with J/B pouches when compared to S/SP pouches could have introduced a bias in favor of the J/B pouch. However, failure-free pouch survival rates after both 10 and 20 years of follow-up are higher for J/B pouches. This difference in survival rate does not refute the potential bias in favor of J/B pouches through medical and surgical innovations that occurred during the time period from when the S/SP was favoured to the period when common construction of the J/B design was introduced. Data on pouches other than J pouches are extremely sparse since the J- design has been widely applied in the past decades. Available literature reports similar and inevitable differences in cohort volume and follow-up period between J and S pouches [4, 13]. Nevertheless, one study reported an increased risk of surgical related complications mainly including mechanical issues in the S pouches when compared to J designed pouches [13]. Although one meta-analysis reported a similar risk for developing pouch failure between S and J pouches, another definition of pouch failure was applied when compared to the definition used in the current study [4]. Moreover, the S/SP cohort of the current study included two S pouches, precluding any reliable comparison to the results of these published studies.

Longitudinal pouch length was reported in 37.5% of patients. Both missing data and variation in (post)operative stage during measurement precluded drawing firm conclusions on optimal pouch length. In the current study, S/SP pouches had a significantly larger pouch size when compared to J/B pouches. Conceptually, S/SP pouches have been constructed larger and presumably dilated over time due to evacuation problems. J/B pouches were routinely constructed with a longitudinal length of ± 10 cm in our tertiary centre, and demonstrated a lower incidence of failure when compared to the larger constructed S/SP pouches. A median longitudinal pouch length of 13.5 cm was found for J and B pouches in either endosopcic, surgical or pouch resection reports. This finding supports the hypothesis of dilatation of pouches after long-term functionality.

A larger proportion of S/SP pouches were constructed for polyposis. Although patients with pouches constructed for polyposis generally have superior long-term outcomes when compared to pouches constructed for UC, differences in pouch failure rates were strongly in favor of J/B pouches [14].

Although surgical techniques surely have improved during and after the study period, the reported median follow-up of 27 years after primary IPAA surgery was longer than currently available literature on long-term outcomes per pouch design [4, 13]. Although these data might be less representable in the current practice, the current study clarified that a sufficient follow-up period is required to gain insight in the very long-term outcomes of various pouch designs.

Confounding variables such as primary sclerosing cholangitis, obesity and acute postoperative anastomotic leakage which could have interfered with results of the current study were missing from patient reports. However, missing data were expected and inevitable due to the studied period of the current study. Both missing data and heterogenuous and small sample sizes in the S/SP cohort specifically precluded more reliable multi-variable or sub-analyses. The difference in surgical skills of the various surgeons constructing pouches within the study period is another bias potentially affecting outcomes. Besides, functional data was not assessed and follow-up data might be incomplete due to the retrospective nature. Follow-up visits were not standardly indicated after pouch surgery for patients in this cohort. However, our hospital is the main referral clinic for pouch-related problems in the Netherlands. Therefore, we expect that the vast majority of patients with PF that underwent pouch-surgery in our hospital were referred back and included in the follow-up of this study.

This study assessed causes for PF for S/SP and J/B designs separately and elaborated on features and outcomes of different pouch designs. PF rates of the J and B pouch were higher when compared to more recently created J pouches, as many surgical and medical innovations occurred the past 20 years. However, long-term results of the J pouch and B-shaped adaptions were far superior when compared to the S and septated pouches. Mechanical outlet issues caused PF in S/SP pouches remarkably more frequently, due to the oversizing in combination with intussusception of pouch-wall and—septa and kinking of the efferent loop. Results of the current study indicate oversizing a pouch should possibly be avoided, as larger designs include a risk of mechanical outlet obstruction. Moreover, Miratashi et al. demonstrated an improved pouch-function in shorter constructed pouch-sizes [15]. We hypothesize that shortage of pressure to overcome resistance of the anal sphincter causes impaired accurate emptying and paradoxal diarrhea in an oversized pouch. The lack of oversizing and outlet obstruction in the J/B pouch group supports this hypothesis. Therefore, we discourage practicing surgeons to consider making larger pouches to reduce defecation frequency. The optimal (J-)pouch size however has to be determined by performing a prospective (randomised) study.

## Supplementary Information

Below is the link to the electronic supplementary material.

Supplementary file1 (TIF 5024 KB)

Supplementary file2 (PDF 212 KB)
